# sitePath: a visual tool to identify polymorphism clades and help find fixed and parallel mutations

**DOI:** 10.1186/s12859-022-05064-4

**Published:** 2022-11-24

**Authors:** Chengyang Ji, Na Han, Yexiao Cheng, Jingzhe Shang, Shenghui Weng, Rong Yang, Hang-Yu Zhou, Aiping Wu

**Affiliations:** 1 Institute of Systems Medicine, Chinese Academy of Medical Sciences and Peking Union Medical College, Beijing, 100005 China; 2 Suzhou Institute of Systems Medicine, Suzhou, 215123 Jiangsu China; 3 School of Life Science and Technology, China Pharmaceutical University, Nanjing, 211100 China

**Keywords:** Phylogenetics, Sequence analysis, Visualization

## Abstract

**Background:**

Identifying polymorphism clades on phylogenetic trees could help detect punctual mutations that are associated with viral functions. With visualization tools coloring the tree, it is easy to visually find clades where most sequences have the same polymorphism state. However, with the fast accumulation of viral sequences, a computational tool to automate this process is urgently needed.

**Results:**

Here, by implementing a branch-and-bound-like search method, we developed an R package named sitePath to identify polymorphism clades automatically. Based on the identified polymorphism clades, fixed and parallel mutations could be inferred. Furthermore, sitePath also integrated visualization tools to generate figures of the calculated results. In an example with the influenza A virus H3N2 dataset, the detected fixed mutations coincide with antigenic shift mutations. The highly specificity and sensitivity of sitePath in finding fixed mutations were achieved for a range of parameters and different phylogenetic tree inference software.

**Conclusions:**

The result suggests that sitePath can identify polymorphism clades per site. The clustering of sequences on a phylogenetic tree can be used to infer fixed and parallel mutations. High-quality figures of the calculated results could also be generated by sitePath.

**Supplementary Information:**

The online version contains supplementary material available at 10.1186/s12859-022-05064-4.

## Background

Identifying polymorphism clades on phylogenetic tree could help detect punctual mutations that are associated with viral function. In viral evolution, fixed mutation, which quickly dominates the viral population since its initial introduction, is accompanied by the formation of polymorphism clades and is usually associated with the shift of key functions [1, 2]. While parallel mutation, which refers to the mutation occurring independently in different polymorphism clades, has a high probability of being the result of natural selection under selective pressure [3].

With visualization tools coloring the tree branches according to a selected site, it is easy to visually find clades where most sequences have the same polymorphism state. However, with the increasing viral sequences in public database, a tool to automate this process is needed. A reasonable algorithm aimed at resolving a given phylogenetic tree into polymorphism clades should consider both the site polymorphism and the tree topology, which is not included in current methods [4, 5].

In this study, we developed an R package named sitePath to resolve tree trunks and identify polymorphism clades per site automatically. Using aligned sequences and an estimated tree as input, sitePath resolves tree trunks by clustering sequences with the same polymorphism state and implements a branch-and-bound-like search method to identify polymorphism clades per site through entropy minimization. The function of Inferring fixed mutation, parallel mutation and visualizing the calculated results are all integrated into the R package.

## Implementation

### Identification of phylogenetic tree trunks

**Fig. 1:**
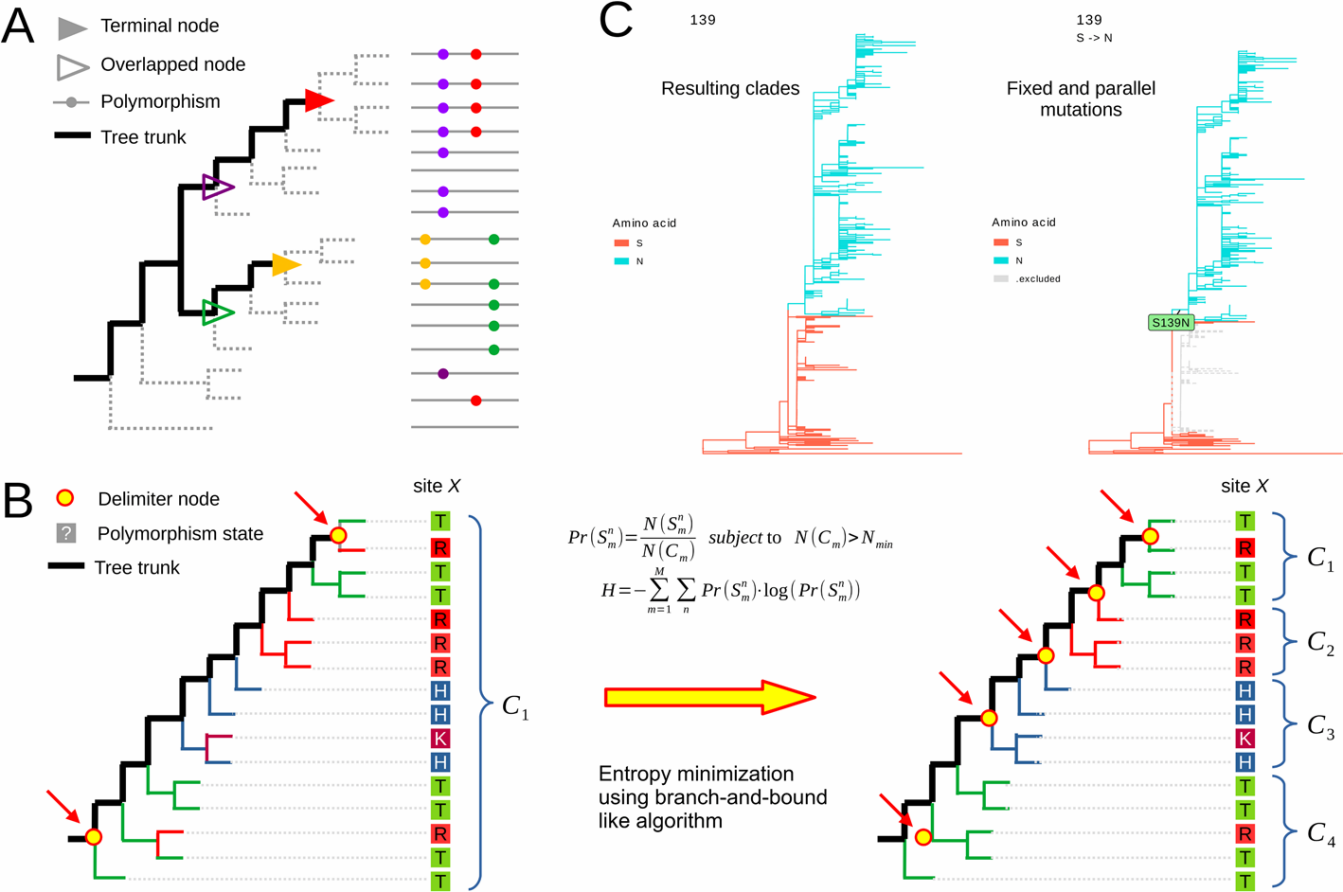
Schematics and demonstration of sitePath. **A** All sequences but one on the upper side are in a monophyletic group with the purple polymorphism, which is also true for the sequences with the green polymorphism; the ancestral nodes of purple and red overlap on tree trunks so only red is retained, which is also the case for the gold and green polymorphisms. **B** For site $X$, the initial entropy ${H}_{0}$ is calculated by clading all sequences as $\left\{{C}_{1}\right\}$; the minimum entropy ${H}_{min}$ is achieved here when the clade set $\left\{{C}_{1}, {C}_{2}, {C}_{3}, {C}_{4}\right\}$ is found. The tree is colored according to the polymorphism state of site $X$ before clading and the dominant polymorphism state after clading. **C** An example of the resulting clades and mutation detection of the ZIKA virus dataset for site 139 of the polyprotein

To resolve tree trunks, sitePath assumes that most descendant sequences of each terminal node have the same polymorphism state. The Treemmer algorithm [6] collapses tree tips and guarantees that the collapsed tree tips are within a monophyletic group. Here, the terminating condition for the Treemmer algorithm is changed to tree tips having the same polymorphism state. However, it is difficult to retrieve a monophyletic group when a few sequences in the group have different polymorphism states than the dominant one (Fig. 1A). The modified Treemmer algorithm would identify a few smaller groups instead of a single monophyletic group. To address this issue, another method similar to CD-HIT [7] is implemented in an attempt to merge the smaller groups. The rule is that the average similarity of the group after merging is no lower than a threshold. The groups of sequences to be merged depend on which site being evaluated. It is therefore impossible to define a universal threshold for all sites. Instead, a universal rule for generating the threshold value is set as the median similarity of the sequences. Furthermore, a polymorphism state has to exist in enough sequences to be valid for defining clades and tree trunk terminals. Thus, the group with the number of sequences less than the predefined ${N}_{\mathrm{min}}$ (the minimum number of sequences to define a valid polymorphism clade) will be treated as outlier and dropped. The tree trunks are found by linking the tree root and the ancestral node of all resulting groups. Only the longest tree trunks are retained if there are overlaps (Fig. 1A).

**Fig. 2:**
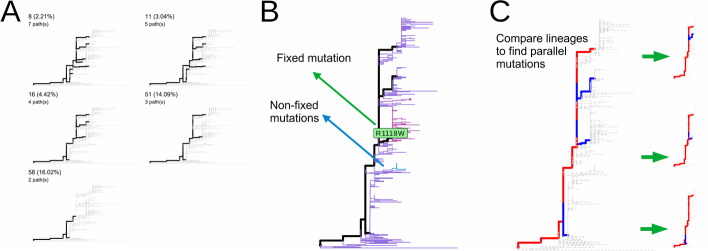
Default ${N}_{\mathrm{min}}$ value and recognition of fixed and parallel mutations. **A** Visualization of phylogenetic pathways using five different ${N}_{\mathrm{min}}$. **B** Fixed and non-fixed mutations were identified along a phylogenetic pathway. **C** The parallel mutation was recognized by comparing mutations between pairs of pathways

The phylogenetic pathways are derived from tree trunks. A phylogenetic pathway can be a combination of tree trunks to describe the ancestral and descendant relationships of clades. A clade closer to the tree root is defined to be ancestral to its neighbor clade. The default ${N}_{\mathrm{min}}$ value depends on the total number of sequences in the input dataset. In sitePath, the default ${N}_{\mathrm{min}}$ is defined as the number of pathways remains the same for a few consecutive ${N}_{\mathrm{min}}$ values. For an input file containing all the available sequences of a virus or a proportional subsampled dataset of it, the default ${N}_{\mathrm{min}}$ is recommended. While for a partial dataset of a virus, sitePath provides a method to help find the best ${N}_{\mathrm{min} }$ (Fig. 2A).

### Branch-and-bound search for polymorphism clades

A branch-and-bound like search method is implemented to minimize the entropy of the polymorphism state across clades. The search is independently performed per site per tree trunk and the site will be referred as site $X$ in this section. The goal is to search for tree trunk nodes delimiting the sequences into such clade set $\left\{{C}_{1}, {C}_{2}, \dots , {C}_{M}\right\}$ that the total entropy $H=-\sum_{\mathrm{m}=1}^{M}\sum_{n}\mathrm{Pr}\left({S}_{m}^{n}\right)\times \mathrm{log}\left(\mathrm{Pr}\left({S}_{m}^{n}\right)\right)$ is minimized (Fig. 1B). Assume in clade ${C}_{m}$ there are $N\left({C}_{m}\right)$ sequences in total and $N\left({S}_{m}^{n}\right)$ sequences with polymorphism state $n$ for site $X$. Then the probability of drawing polymorphism state $n$ in ${C}_{m}$ is $Pr\left({S}_{m}^{n}\right)=N\left({S}_{m}^{n}\right)/N\left({C}_{m}\right)$. The search starts with initial entropy ${H}_{0}$ by treating all sequences on the tree trunk as a single clade delimited by the tree root and the terminal node. Then, the search branches to explore new clade sets through iterations to find the goal clade set with the minimum entropy ${H}_{\mathrm{min}}$.

A breadth-first searching strategy is used, meaning that all possible clade sets branched from the previous clade set are evaluated when proceeding with the search. All $N({C}_{m})$ of a newly branched clade set is constrained to be greater than the predefined ${N}_{\mathrm{min}}$ otherwise it will become inactive in later iterations. For each iteration, all the newly branched and other active clade sets are compared to find the temporary best solution with the lowest $H$ value. This is because $H$ is not monotonically decreasing in the search towards the goal clade set. The search is terminated when the temporary best solution stays the best after a few iterations. It is set as the total number of nodes of the tree trunk by default and can be folded by user to elongate the search.

### Fixed and parallel mutation

**Fig. 3:**
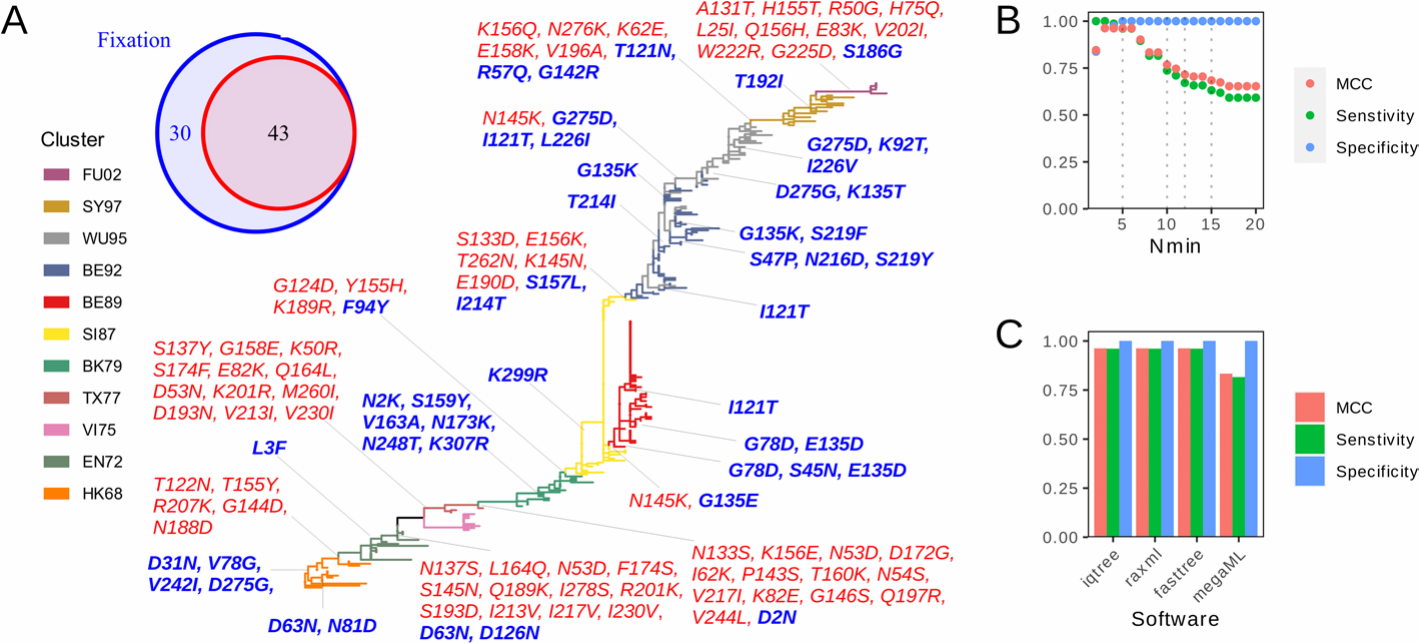
Performance evaluation of sitePath. **A** Recognition of fixed mutations in influenza A H3N2 with sitePath. Mutations are shown for all 43 antigenic cluster-determining sites [1] in red and an additional 30 fixed sites detected by sitePath are colored in blue. **B**, **C** Changes in specificity, sensitivity and MCC against ${N}_{\mathrm{min}}$ (**B**) and different phylogenetic tree-estimating software (**C**)

The result can be further used to find fixed and parallel mutations in phylogenetic pathways (Fig. 2). Fixed mutation was found by comparing the dominant polymorphism state between adjacent clades (Fig. 2B), while parallel mutation was found when a variant was observed on multiple tree trunks (Fig. 2C). The performance (Fig. 3) of detecting fixed mutations is tested on an influenza A H3N2 antigenic drifting dataset [1]. For visualization, sitePath provides functions based on the R package ggtree [8]. As an example, a ZIKA virus dataset (see Additional file 1 for NCBI Accessions) is included in sitePath package. The result of its site 139 of the polyprotein, which was reported to be associated with enhanced virulence [2], is shown in Fig. 1C. Moreover, sitePath was used on SARS-CoV-2 to find fixed and parallel mutations, which were found to be potentially adaptive for the virus [9].

### Computation time complexity

Tree trunk and clade identification approximately share the same algorithm time complexity of $O\left(SL/{N}_{\mathrm{min}}\right)$, where $S$ is the total number of sequences and $L$ is the length of the sequence alignment. To save time, the sites that are completely conserved or have too many ambiguous characters or gaps are ignored.

## Results

### Performance of sitePath finding fixed mutations

We tested sitePath on an influenza A H3N2 dataset with fixed mutations, which matches the antigenic shift of the virus [1]. In this dataset, 43 fixed mutations were identified as antigenic-determined sites based on experimental work. All 43 fixed mutations in Smith et al.’s work were detected by sitePath. An extra set of 30 previously unreported fixed mutations was found (Fig. 3A). Note that these 30 additional mutations occurred in inter- or intra-antigenic clusters, which may explain why these mutations were not listed in Smith et al.’s report.

The robustness of sitePath against the sole parameter ${N}_{\mathrm{min}}$ was also tested using Smith et al.’s dataset. Specificity, sensitivity and MCC (Matthews Correlation Coefficient) are relatively high for smaller ${N}_{\mathrm{min}}$ values (Fig. 3B). Sensitivity and MCC decrease with higher ${N}_{\mathrm{min}}$ values because the thresholds were too strict for sitePath to identify smaller clades. The impact of different tree-estimating software, including IQ-TREE [10], FastTree [11], and RaxML [12] was also tested. They were all set to use the GTR substitution model with the GAMMA model of rate heterogeneity for MLE (Maximum Likelihood Estimation) method. Sensitivity, specificity and MCC were stable, reaching 0.95, 1 and 0.95 respectively for all three software (Fig. 3C). However, we also noticed that sensitivity and MCC decreased to 0.8 for MEGAX [13]. The models for other software were not available for the MLE method of MEGAX, and the JTT substitution model was used instead. The tree topology inferred by MEGAX partly disagrees with the antigenic cluster. A few fixed mutations were not dominant in the clade because of the topology disagreement, hence missed by sitePath as true positive. The source code for the performance test can be found at https://github.com/wuaipinglab/sitePath_assessment.

## Conclusions

To summarize, sitePath is a tool that attempts to cluster adjacent sequences on the phylogenetic tree using polymorphism per site per tree trunk. The branch-and-bound-like search method is used to minimize the polymorphism state across the clusters. The clustering result can be used to detect mutations with fixation or parallel patterns on the phylogenetic tree and all the results can be visualized accordingly.

The application of sitePath in viral sequences has been fully tested. In theory, sitePath could also be used in data sets with complex genomes such as prokaryotic and eukaryotic sequences. The computational consumption of dealing with complex sequences could also benefit from the neglect of conserved sites. However, considering the large size and more complex functional complexity of prokaryotic and eukaryotic sequences, it is better to apply sitePath in specific gene segments rather than the whole genomes.

## Availability and requirements

Project name: sitePath

Project home page: https://github.com/wuaipinglab/sitePath

Operating systems: Cross-platform

Programming language: R, C++

License: MIT License

Any restrictions to use by non-academics: License needed

## Supplementary Information

**Additional file 1**: NCBI Accession for the ZIKA virus dataset.

## Data Availability

The sequences for ZIKA virus is publicly available at NCBI protein database (https://www.ncbi.nlm.nih.gov/protein/) and the accession numbers can be found in Additional file 1. The dataset used for the performance test is available from Smith et al.’s [1] study.
